# Tracking respiratory mechanics around natural breathing rates via variable ventilation

**DOI:** 10.1038/s41598-020-63663-8

**Published:** 2020-04-21

**Authors:** Samer Bou Jawde, Allan J. Walkey, Arnab Majumdar, George T. O’Connor, Bradford J. Smith, Jason H. T. Bates, Kenneth R. Lutchen, Béla Suki

**Affiliations:** 1 Department of Biomedical Engineering, Boston University, Boston, MA USA; 2 Department of Medicine, Pulmonary, Allergy, Sleep, & Critical Care Medicine, Boston University, Boston, MA USA; 3 Department of Bioengineering, University of Colorado Denver | Anschutz Medical Campus, Aurora, CO USA; 4 Pulmonary/Critical Care Division, University of Vermont, Burlington, VT USA

**Keywords:** Respiration, Respiratory distress syndrome, Preclinical research

## Abstract

Measuring respiratory resistance and elastance as a function of time, tidal volume, respiratory rate, and positive end-expiratory pressure can guide mechanical ventilation. However, current measurement techniques are limited since they are assessed intermittently at non-physiological frequencies or involve specialized equipment. To this end, we introduce ZVV, a practical approach to continuously track resistance and elastance during Variable Ventilation (VV), in which frequency and tidal volume vary from breath-to-breath. ZVV segments airway pressure and flow recordings into individual breaths, calculates resistance and elastance for each breath, bins them according to frequency or tidal volume and plots the results against bin means. ZVV’s feasibility was assessed clinically in five human patients with acute lung injury, experimentally in five mice ventilated before and after lavage injury, and computationally using a viscoelastic respiratory model. ZVV provided continuous measurements in both settings, while the computational study revealed <2% estimation errors. Our findings support ZVV as a feasible technique to assess respiratory mechanics under physiological conditions. Additionally, in humans, ZVV detected a decrease in resistance and elastance with time by 12.8% and 6.2%, respectively, suggesting that VV can improve lung recruitment in some patients and can therefore potentially serve both as a dual diagnostic and therapeutic tool.

## Introduction

Respiratory resistance (*R)* and elastance (*E*) in mechanically ventilated patients relate to disease severity and progression, and patient response to treatment or changes in ventilator settings^1–3^. The manner in which *R* and *E* vary with frequency can reflect lung heterogeneity, a sensitive indicator of pathology^4^. Thus, a technique that can continuously and reliably assess *R* and *E* at physiological frequencies and tidal volumes (*V*_*T*_’s) could advance the treatment of mechanically ventilated patients. For example, increases in *R* can reveal bronchospasm^5^ or a blocked endotracheal tube^6,7^, while increases in *E* can reflect pulmonary edema and alveolar derecruitment^8,9^. Tracking continuously *R* and *E* including their frequency dependencies could improve patient management via detection of airway obstruction in asthma, or optimization of mechanical ventilator settings for preventing ventilation-induced lung injury^10–15^.

Nevertheless, estimates of *R* and *E* are rarely performed in clinical practice^16^, likely due to current strategies of intermittent end inspiratory occlusion measurements that require deep patient sedation or paralysis or the need for specialized equipment^10,17^. In order to adjust ventilation settings, clinicians are currently guided by gas exchange parameters, breath-to-breath peak pressures, and plateau pressures^16,18–20^. However, these measures lack the ability to reveal whether abnormalities in mechanics are related to altered *R* or *E* and often require a heavily sedated or paralyzed patient^9,21^. The limitations in measuring *R* and *E* are not only present in the clinical setting but also extend to research settings in animals, where for example, commercial ventilators have to pause ventilation and deliver non-physiological waveforms to assess respiratory mechanics.

Here, we report the development of a novel approach – termed ZVV – to provide continuous measurement of *R* and *E* at physiologically relevant frequencies and tidal volumes without requiring additional ventilator equipment, patient manipulations, or ventilation interruption. ZVV exploits a relatively new approach to mechanical ventilation known as Variable Ventilation (VV). VV was introduced in 1996 by Lefevre *et al*.^22^ with the notion that natural breathing varies on a breath-by-breath basis, but mechanical ventilation eliminates this variability. They showed that introducing breath-by-breath variability in tidal volume into mechanical ventilation improved gas exchange in an animal model of acute lung injury. Current clinical ventilators, however, only utilize conventional ventilation (CV) in which both the delivered tidal volume and respiratory rate are fixed. On the other hand, VV provides physiological variation in tidal volumes and breathing frequencies on a breath-by-breath basis, which, in a mathematical model predicted to improve recruitment of atelectatic lung regions^23^, confirmed later by many animal studies^24–37^. Hence, with a distinct amplitude and frequency at each breath, the lung is exposed to multiple frequencies and tidal volumes surrounding those of natural breathing without the requirement of additional equipment.

We hypothesized that the variability present in VV can be utilized to assess dependencies of *R* and *E* on both tidal volume and frequency. To this end, we analyzed pressure-flow data previously collected during VV from patients with mild acute respiratory distress syndrome (ARDS) and mechanically ventilated mice under controlled experimental conditions before and after lung lavage as an ARDS model. Finally, we validated ZVV’s accuracy with computational modeling.

## Methods

### Measurement of respiratory impedance (Z) via Variable Ventilation (VV)

**Figure 1:**
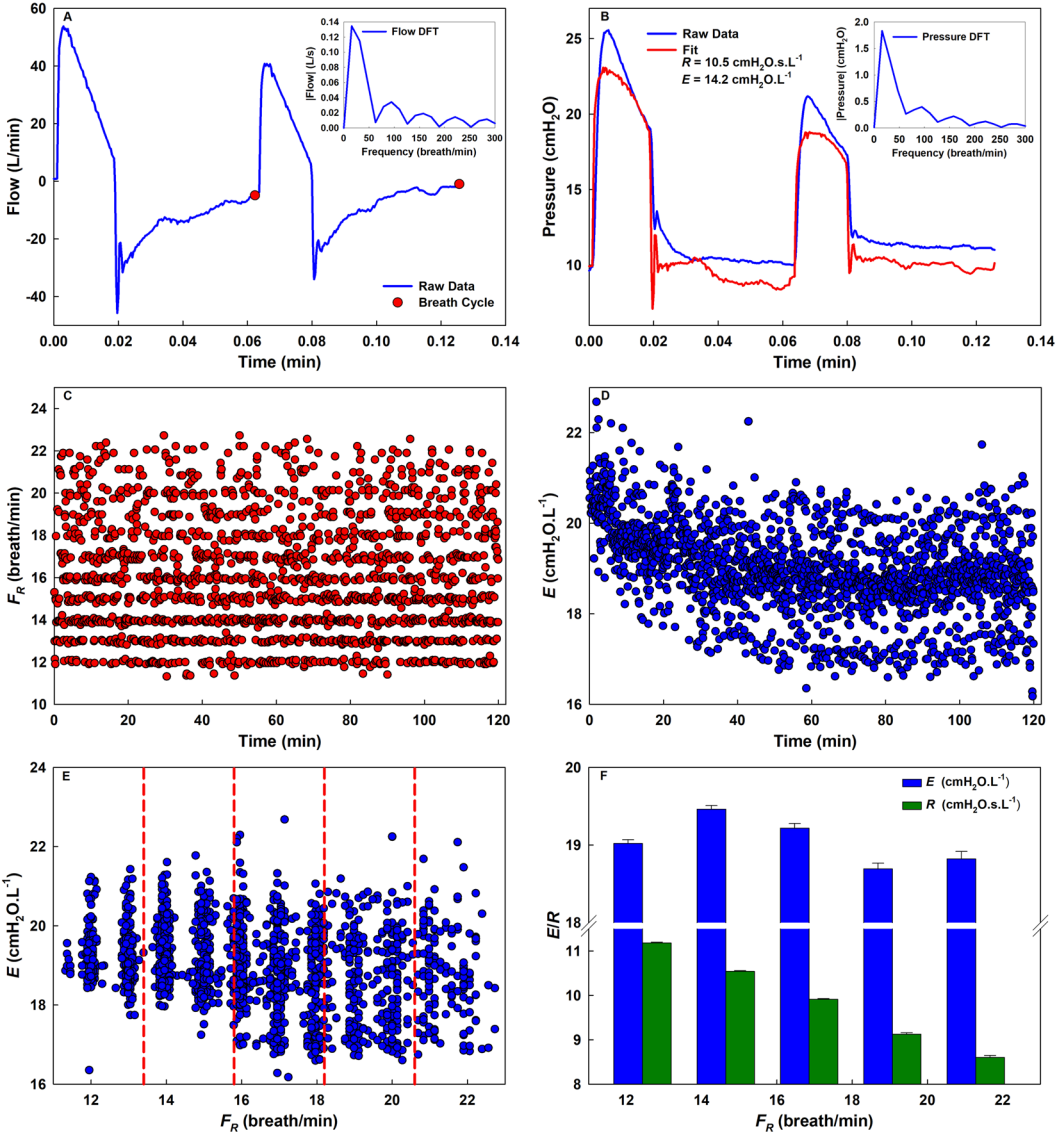
The ZVV approach. Airway opening flow (**A**) and pressure (**B**) time series of two consecutive cycles with different *V*_*T*_’s from a patient. Notice that the two breaths are different due to the statistically defined pattern of breaths in VV. The flow is used to detect breath cycles as shown in the 2^nd^ cycle flow data (red solid circles). The corresponding Discrete Fourier Transforms (DFTs) are shown in the insets. Using the transforms, *R* and *E* are computed from the complex ratio of pressure and flow, Z, as the real part and −2π*F*_*R*_ times the imaginary part, respectively. The pressure fit tracing is also plotted (red line). (**C**) *F*_*R*_ and (**D**) *E* for every breath as a function of time. (**E**) *E* plotted as a function of *F*_*R*_ can be divided into five equally-spaced frequency intervals separated by red dashed lines and (**F**) binned into histograms showing the mean and standard error at every bin center. Similarly, *R* is binned as a function of *F*_*R*_. (**D**) shows a decrease in *E* with time, while (**F**) shows that *E* is mainly constant unlike *R* which decreases with frequency.

The ZVV (*Z* + *VV*) approach is introduced in Fig. 1 showing recordings of airway opening flow ($\dot{V}$, Fig. 1A) and pressure (*P*, Fig. 1B) tracings of two consecutive cycles from a patient. For the 2^nd^ cycle, the respiratory impedance (*Z*) is calculated by dividing the Discrete Fourier Transform (DFT) of *∆P*, the pressure difference between *P* and positive end-expiratory pressure (PEEP), with that of $\,\dot{V}$. Respiratory resistance (*R)* and elastance (*E)* are evaluated at the spontaneous breathing frequency (*F*_*R*_) as the real part of *Z* and the imaginary part of *Z* multiplied with −2π*F*_*R*_, respectively (Fig. 1B). Calculating *F*_*R*_ (Fig. 1C) and *E* (Fig. 1D) for each breath, *E* is plotted as a function of *F*_*R*_ (Fig. 1E). By dividing the data in Fig. 1E into 5 equally-spaced *F*_*R*_ bins, a final graph of the mean ± standard error is plotted (Fig. 1F). A similar process is carried out for *R* (Fig. 1F). In order to avoid systematic and random errors corresponding to faulty breath detection and non-physiological values, the data are filtered by setting a minimum and maximum *F*_*R*_, *R* and *E* and excluding values outside the range. Then, for each bin, *R* and *E* values that are 2 standard deviations away from the mean are removed. The means and deviations of *R* and *E* are then recomputed.

Similarly, *R* and *E* can be binned as a function of *V*_*T*_. Furthermore, changes in mechanical function with respect to different time periods (T) can be obtained by separating the total data into different time segments. To capture the dependence of *R* and *E* on *V*_*T*_, *F*_*R*_, and T, they are plotted against various bin combinations within each time segment.

### Human clinical trial

The human data analyzed in this study, was previously collected from five male patients with mild ARDS (see Supplemental Table S1 for biographic data). The protocol to ventilate patients with VV was approved by the FDA (https://clinicaltrials.gov/ct2/show/NCT01083277) and the IRB of Boston University School of Medicine and all methods were carried out in accordance with relevant guidelines and regulations for human subjects. Additionally, informed consent was obtained from all subjects or a family member in case the patient was not able to sign the consent form. The patients were mechanically ventilated using a modified ventilator capable of delivering VV (Puritan Bennett 840 Ventilator, Covidien. Ireland, Dublin) with a graphical user interface to monitor outputs. A custom-designed computer program (LabVIEW, National Instruments, Austin, TX) was used to determine the ventilation settings. The ventilation was divided into two sections, one with CV and another with VV delivered in random order with a 1 h washout period between them using CV. However, ZVV requires VV, and thus, for the scope of this study, only the VV data is reported. All patients received sedatives with the goal sedation of Riker level 3 or 4. At the beginning of the VV session, the respiratory therapist set up the baseline ventilation parameters including tidal volume (*V*_*T,B*_) and respiratory rate (*F*_*B*_) based on biometric data and patient condition. A range of tidal volumes was calculated such that the peak inspiratory pressure (PIP) was uniformly distributed within a range of ±30% around the mean PIP corresponding to *V*_*T,B*_^23^. This was achieved by first delivering 3 cycles with a *V*_*T*_ = 1.4*V_*T,B*_ and recording *P* and $\dot{V}$ in order to obtain a partial pressure-volume curve. For each VV cycle during the protocol, a PIP was randomly chosen from this distribution and the pressure-volume curve was used to compute the corresponding *V*_*T*_ to be delivered. To maintain constant minute ventilation on a cycle-by-cycle basis, *F*_*R*_ was computed for each cycle as *F*_*R*_ = *F*_B_*V_*T,B*_/*V*_*T*_. Finally, for patient comfort, the inspiratory time was allowed to vary only by 10%.

*P* and $\dot{V}$ were recorded (50 Hz) by the ventilator and saved for off-line processing. The $\dot{V}$ tracing was used to determine the beginning and end of every breath, from which *V*_*T*_ and *F*_*R*_ were computed. The recordings were then split into two equal time periods, and the ZVV analysis described above was separately carried out on both the 1^st^ (T1) and 2^nd^ (T2) period. *R* and *E* in each section were grouped according to 5 frequency bins allowed by the cycle-by-cycle fluctuations in *F*_*R*_ and plotted for every patient as a function of *F*_*R*_. The full set of data was also binned into a low *V*_*T*_ (*V*_*T,1*_) and high *V*_*T*_ (*V*_*T,2*_) with the mean *V*_*T*_ separating the two *V*_*T*_ bins. Since the recorded data length varied between patients, the time analyzed varied as well: per period, the time interval ranged from 23 to 64 minutes with the number of breaths between 200 and 900.

### Mouse experiments

The experimental protocol was approved by the Institutional Animal Care and Use Committee of Boston University and all methods were carried out in accordance with relevant guidelines and regulations for the care and use of animals. The experimental setup was described previously^38^. However, utilizing the data for ZVV is presented here for the first time. Briefly, mice (C57BL/6, n = 5, 29.8 ± 2.0 g) were anaesthetized, tracheostomized, and ventilated (flexiVent Legacy, Scireq, Montreal, CA) at *V*_*T,B*_ = 8 ml/Kg using VV with a specific mouse-optimized *V*_*T*_ distribution^24^. A differential pressure transducer (Biopac Systems, Model TSD160A) recorded pressure drop across a lab-designed flow sensor^38^, while a gauge pressure transducer (WPI, 07B PNEU05) was connected distal to the sensor to measure airway opening pressure. The signals were digitized (WPI, DataTrax) at 500 Hz. After delivery of 10 min VV, mice were disconnected from the ventilator and the lungs were lavaged with 0.1 ml warm phosphate-buffered saline instilled into the trachea. VV was then resumed with the same settings for 20 min. The purpose of the lavage was to demonstrate ZVV’s ability to capture changes in *R* and *E* due to ARDS. To assess respiratory mechanics using the ventilator via the standard forced oscillation technique (FOT, see Supplement A for details)^39^, at the end of each VV session, the ventilator delivered 4 optimal ventilator waveform (OVW, see Supplement B for details)^40^ perturbations separated by 12 seconds of CV.

ZVV was carried out both before and after lavage. The periods T1 and T2 included the first and last 5 minutes of VV, respectively. In addition, using all the VV time data before and after lavage separately (pooling T1 and T2), the mean values of *R*, *E*, and *F*_*R*_ were calculated. The evaluated mean of *F*_*R*_ was used to calculate the equivalent *R* and *E* from the OVW data. Specifically, the data obtained from OVW was used to fit the constant-phase model written as^41,42^:

1 $$Z={R}_{N}+{I}_{aw}j+\frac{G-jH}{{\omega }_{n}^{\alpha }}$$

where *R*_*N*_, *I*_*aw*_, *G*, and *H* are the Newtonian resistance, airway inertance, tissue damping, and tissue elastance, respectively, $\alpha =\frac{2}{\pi }{\rm{atan}}\left(\frac{H}{G}\right)$ and $\omega$ is the circular frequency (rad/s). Note that the circular frequency $\omega$ is normalized with ${\omega }_{0}=1$ radian so that ${\omega }_{n}=\frac{\omega }{{\omega }_{0}}$ is used in Eq. (1) in order to obtain meaningful units for *G* and *H*^43^. For the range of frequencies applied, $\,{I}_{aw}\approx 0$. Thus, the equivalent 2-element resistance (*R*) and elastance (*E*) at the mean normalized circular frequency ${\omega }_{m}$ corresponding to the evaluated mean of *F*_*R*_ are written as:

2 $$R={R}_{N}+\frac{G}{{\omega }_{m}^{\alpha }}$$

3 $$E=H{\omega }_{m}^{1-\alpha }$$

These values obtained from 4 OVW recordings were averaged and compared to the ZVV-derived ones.

### Computational modeling

The calculation of respiratory impedance (*Z*) with Fourier analysis requires that the measured system is in a steady state. However, since both breathing frequency *F*_*R*_ and tidal volume *V*_*T*_ vary from cycle to cycle during VV, the mechanical state of the respiratory system is also affected by transients. To test how such transients influence the estimated values of respiratory resistance (*R*) and elastance (*E*) using the ZVV approach, a linear viscoelastic Kelvin body was utilized to represent the lung^44^.

The mechanical model consisted of a dashpot (*R*_*1*_) in series with a spring (*E*_*1*_) and both in parallel with another spring (*E*_*2*_). The parameter values (*R*_*1*_ = 60 cmH_2_O.s.L^−1^, *E*_*1*_ = 20 cmH_2_O.L^−1^, and *E*_*2*_ = 5 cmH_2_O.L^−1^) were selected so as to replicate the magnitudes of *R* and *E* observed in the human data.

The equivalent resistance (*R*_*k*_) and elastance (*E*_*k*_) of the Kelvin body can be computed as follows^45^:

4 $${R}_{k}=\frac{{R}_{1}{E}_{1}^{2}}{{R}_{1}^{2}{\omega }^{2}+{E}_{1}^{2}}$$

5 $${E}_{k}=\frac{{R}_{1}^{2}{E}_{1}{\omega }^{2}+{R}_{1}^{2}{E}_{2}{\omega }^{2}+{E}_{1}^{2}{E}_{2}}{{R}_{1}^{2}{\omega }^{2}+{E}_{1}^{2}}$$

Using a minimum and a maximum *F*_*R*_, and a sequence of *V*_*T*_*’s* similar to those in the human experiments, a time series of simulated VV was constructed by stitching the individual cycles together each having a different volume amplitude (*V*_*o*_) and *F*_*R*_ creating a volume signal (*V*) of the form:

6 $$V(t)=-\frac{{V}_{o}}{2}\,\cos (2\pi {F}_{R}(\Delta t))+\frac{{V}_{o}}{2}$$

where $\Delta t$ represents the difference in time from the onset of each breath cycle. The flow ($\dot{V}$) was computed as the volume trace differentiated with respect to time. Next, the pressure difference (*∆P*) with respect to the positive-end expiratory pressure (PEEP) was obtained for each breath using the analytical solution of the Kelvin body given by:

7 $$\Delta P(t)=P(t)-PEEP=\Delta {P}_{i}{e}^{-\frac{{E}_{1}}{{R}_{1}}\Delta t}+\frac{{V}_{o}}{2}({E}_{2}-{E}_{k})(1-{e}^{-\frac{{E}_{1}}{{R}_{1}}\Delta t})+{R}_{k}\dot{V}+{E}_{k}V$$

where *∆P*_*i*_ is the value of *∆P* at the start of the current breath. Note that due to the variable nature of the ventilation, the pressure difference includes both steady-state and transient pressure contributions from previous breaths. In particular, the last 2 terms in Eq. (7) are the conventional resistive and elastic pressure drops respectively whereas the first 2 terms represent the transients.

In order to account for the transient effects, we approximate the transient part of *∆P*, the first 2 terms in Eq. (7) denoted by *∆P*_*T*_, to vary linearly from the beginning to the end of each breath as follows:

8 $$\Delta {P}_{T}(t)=2\pi {F}_{R}(\Delta {P}_{f}-\Delta {P}_{i})t$$

where *∆P*_*f*_ is the value of *∆P* at the end of the breath. Finally, the estimated steady-state pressure difference (*∆P*_*s*_) of the current breath is computed as:

9 $$\Delta {P}_{s}(t)=\Delta P(t)-\Delta {P}_{T}(t)$$

The concept behind this correction is that in the steady-state, the end of each breath should be the same; thus, any variation in pressure at the end of the breath is due to transients. While these transients might take different functional forms (e.g., not necessarily exponential as in the Kelvin body model), a linear approximation is the simplest. It is worth mentioning that for a pressure-controlled ventilation, a steady-state volume can be estimated in a similar fashion for the correction of transients.

The ZVV procedure was carried out to reconstruct the Kelvin body’s frequency dependent *Z* from the simulated VV data. To observe the effects of the transients and breath-to-breath variations in *F*_*R*_/*V*_*T*_, a secondary binning was carried on both *R* and *E*. Specifically, after binning the data across *F*_*R*_, the *R* and *E* spectra were further binned based on the time difference in periods between consecutive breaths (∆T_b_). Then the correction method to estimate a steady-state pressure difference (*∆P*_*s*_) between absolute airway pressure and the PEEP in order to minimize the effects of transients was applied. To test the robustness of this correction (Eqs. (8) & (9)), starting from the baseline simulation (number of breath = 500 breaths, 10 ≤ *F*_*R*_ ≤ 20 breath/min, minute ventilation = 7.5 L/min), an additional 14 sets of simulations were carried out in which only one parameter was changed at a time. For each simulation, the mean values of *R* and *E* using both *∆P*_*s*_ and *∆P* were computed and grouped into 8 frequency bins and the errors with respect to the Kelvin body (Eqs. (4) & (5)) were calculated.

### Statistical Analysis

All data analyses and statistical tests were performed using MATLAB R2018b (MathWorks, CA). For the human data, to account for the effects of *F*_*R*_, T, and any interaction between these factors, a 2-way Anova was applied to the data set. For the Anova, the *F*_*R*_ bins were categorized from 1 to 5 representing the lowest and largest frequency bin centers, respectively, while T was categorized to 1 and 2 corresponding to T1 and T2, respectively. For mice, a 3-way Anova was used with the additional effect of lavage. Following the 3-way Anova, a multiple comparison test (Tukey’s honestly significant difference) was used to separate the effects of lavage within T. Paired t-tests were used to compare values of *R* and *E* from VV and OVW. Rank-sum tests were used to compare *V*_*T*_ dependences. Statistical significance was accepted at p < 0.05.

### Notation of prior abstract publication/presentation

Part of this work was given as a poster presentation at the 2018 annual meeting of the Biomedical Engineering Society (October 17–20, Philadelphia, PA) and as an oral presentation at the 2019 annual meeting of the American Thoracic Society (May 17–22, Dallas, TX).

### Clinical Trial Registration Number

NCT01083277.

## Results

### Human Clinical Trial

**Figure 2:**
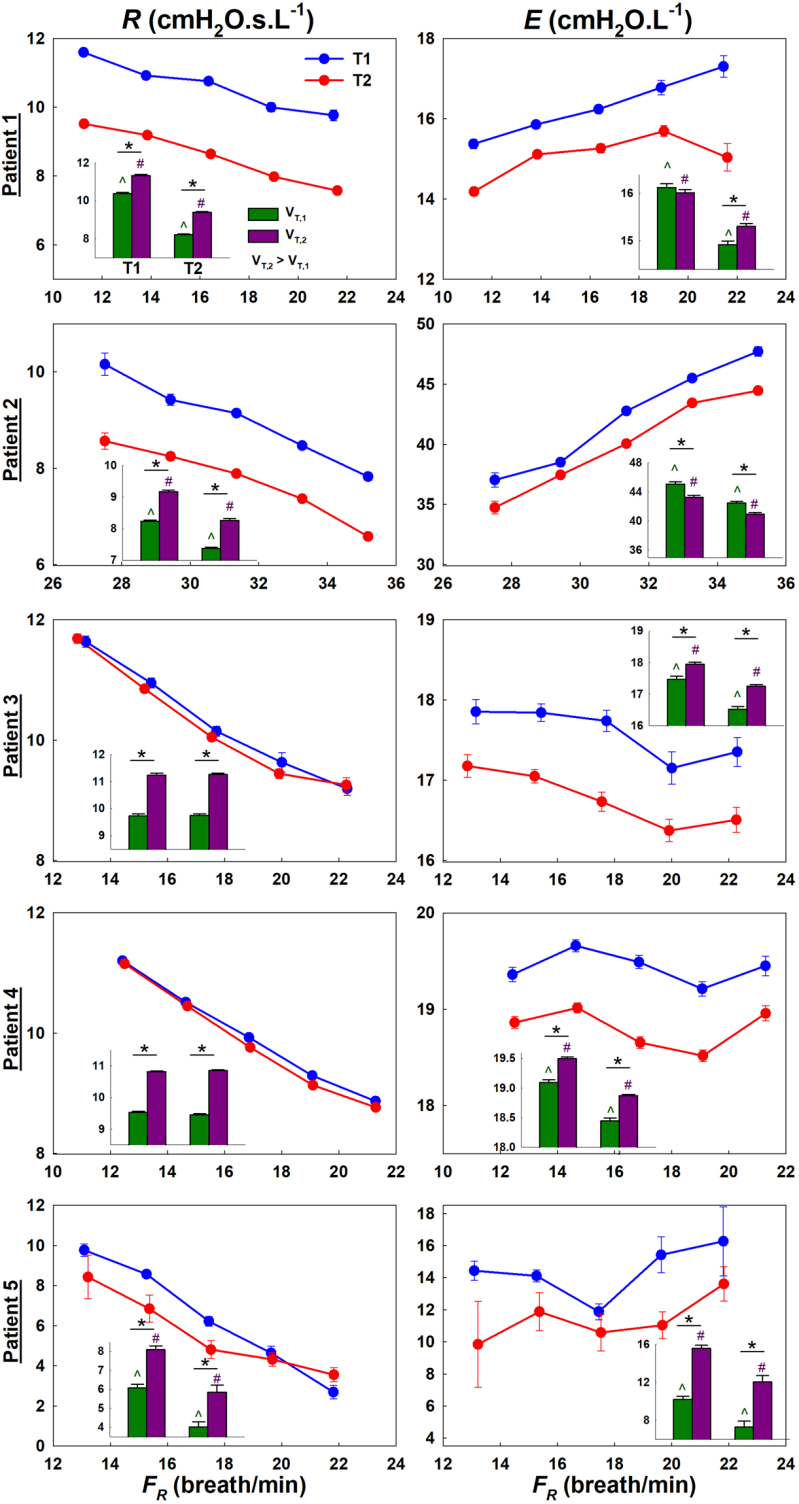
Human ZVV analysis results showing the binned means and standard errors of *R* and *E* as a function of *F*_*R*_ (solid lines) and *V*_*T*_ (bar chart insets) for both the first and second halves of the time recording (T1 and T2). Notice that the lowest *F*_*R*_ in patient 2 was greater than the highest *F*_*R*_ in all other patients. Note also that the error bars are often smaller than the symbols. (*): significance between *V*_*T*_ within same T. (^) and (#): significance from T1 to T2 for *V*_*T,1*_ and *V*_*T,2*_ (*V*_*T,2*_ > *V*_*T,1*_).

Applying ZVV to all patients made it possible to construct the dependence of *R* and *E* on *F*_*R*_ and *V*_*T*_ (Fig. 2). ZVV revealed both general trends as well as patient specific results within the population. For instance, *R* and *E* decreased from T1 to T2 in all patients while the amount of drop varied among patients. While *R* decreased with increasing *F*_*R*_, changes in *E* were patient specific. *R* increased with *V*_*T*_ in all patients in both T1 and T2, but changes in *E* were again patient specific with patients 3–5 displaying an increase during both T1 and T2. The full statistics, summarized in Table 1, show that in all 5 patients, *E* decreased significantly with T (p < 10^−5^). In 4 patients, *R* also decreased significantly (p < 0.005) from T1 to T2. In all 5 patients, *F*_*R*_ dependence of *R* and *E* was statistically significant, but the interaction between *F*_*R*_ and T was significant only in 2 patients.

**Table 1:** Summary of human acute lung injury results.

		T1			T2			T1 Vs. T2	p-values		
Patient	Time Studied (min)	Number of Breath	Mean	Standard Error	Number of Breath	Mean	Standard Error	(T2-T1)/T1	*F*_*R*_	T	T*F_*R*_
Respiratory Resistance (cmH_2_O.s.L^−1^)											
1	107	677	10.8	0.05	688	8.9	0.03	−18.2%	**<10**^**−80**^	**<10**^**−100**^	**0.022**
2	47	639	8.8	0.05	608	7.6	0.04	−13.2%	**<10**^**−100**^	**<10**^**−50**^	0.26
3	60	327	10.58	0.063	419	10.56	0.054	−0.2%	**<10**^**−100**^	0.4	0.71
4	120	895	10.13	0.028	888	10.07	0.028	−0.5%	**<10**^**−100**^	**<10**^**−5**^	0.060
5	129	482	7.2	0.15	203	4.9	0.23	−31.8%	**<10**^**−50**^	**0.0049**	**0.019**
**Respiratory Elastance (cmH**_**2**_**O.L**^**−1**^**)**											
1	107	681	16.1	0.06	683	15.0	0.05	−6.4%	**<10**^**−10**^	**<10**^**−30**^	**0.0005**
2	47	637	43.6	0.20	603	41.0	0.17	−6.0%	**<10**^**−150**^	**<10**^**−30**^	**0.020**
3	60	326	17.7	0.07	423	16.9	0.06	−4.6%	**<10**^**−5**^	**<10**^**−10**^	**0.80**
4	120	900	19.5	0.03	898	18.8	0.03	−3.3%	**<10**^**−10**^	**<10**^**−30**^	**0.080**
5	129	487	13.8	0.30	199	11.6	0.52	−15.8%	0.0043	**<10**^**−5**^	**0.34**

Note that the differences in the number of breaths used to evaluate *R* and *E* are due to removing absolute values larger than the mean plus two standard deviations for each parameter independently.

### Mouse experiments

**Figure 3:**
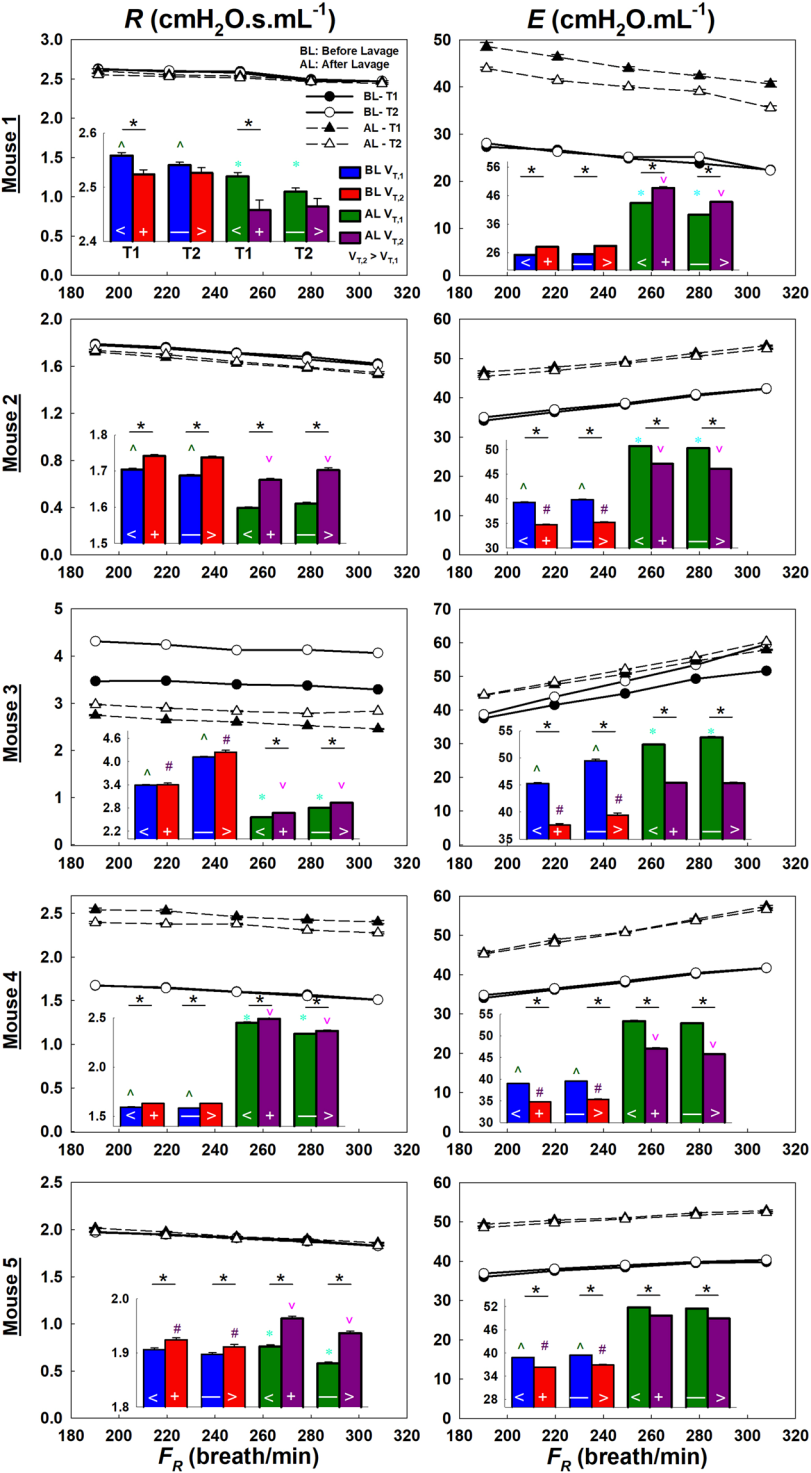
Mouse ZVV analysis results showing the means and standard errors of *R* and *E* as a function of *F*_*R*_ and *V*_*T*_ (bar charts insets) for T1 and T2 before (solid lines) and after (dashed lines) lavage. With respect to *F*_*R*_, *R* showed a decrease in all mice, while *E* increased in 4 out of 5 mice before and after lavage. With respect to *V*_*T*_, expect for mouse 1, *R* increased while *E* decreased significantly with increasing *V*_*T*_ both in T1 and T2 before and after lavage. Following lavage, *E* increased in all mice while *R* was subject specific either decreasing or increasing. However, the effect of T on *E* before and after lavage was different (Supplementary Table S3). Although the percent changes were small (<10%), they were statistically significant in 4 out of 5 mice. (*): Significance between *V*_*T*_ within same T. (^) and (#): significance between T1 and T2 for *V*_*T,1*_ and *V*_*T,2*_ before lavage. (*) and (^): significance between T1 and T2 for V_T,1_ and V_T,2_ after lavage. (<) and (+): significance before and after lavage during T1 for V_T,1_ and V_T,2_. (—) and (>): significance before and after lavage during T2 for V_T,1_ and V_T,2_.

Compared to the human data, ZVV under experimentally controlled conditions revealed qualitatively similar as well as mouse specific results (Fig. 3 & Supplementary Table S2). Interestingly, lavage reversed the effect of T: before lavage, *E* increased, while following lavage, it decreased from T1 to T2 (Supplementary Table S3).

Next, we compared *R* and *E* from ZVV to those obtained using the OVW-based FOT (Supplements A&B)^39,40^. The means and standard errors of *R* and *E*, in units of cmH_2_O.s.ml^−1^ and cmH_2_O.ml^−1^, respectively, calculated using ZVV were 2.31 ± 0.36 and 38 ± 3.1 before lavage and 2.23 ± 0.18 and 49 ± 1.9 after lavage. The corresponding values obtained from OVW at the mean *F*_*R*_ were 0.93 ± 0.33 and 35 ± 0.8 before lavage and 0.96 ± 0.17 and 48 ± 0.7 after lavage, respectively. While *E* from ZVV and OVW did not differ, the difference in *R* was significant (p = 0.036 and p = 0.002 before and after lavage, respectively).

### Computational Modeling

**Figure 4:**
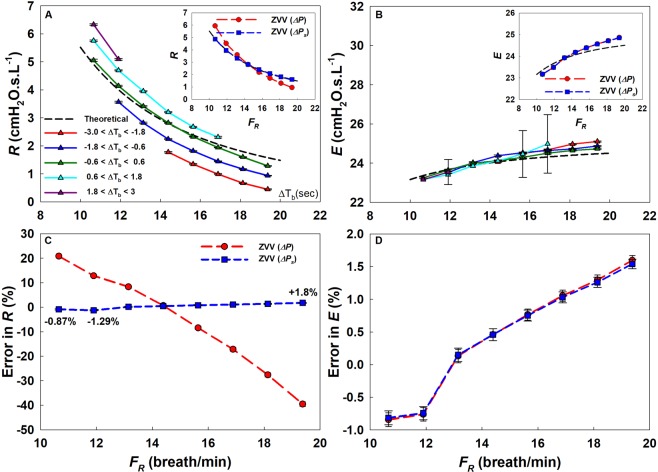
Kelvin body simulations. *R* (**A**) and *E* (**B**) as a function of *F*_*R*_ and ∆T_b_, the difference in the periods between consecutive breaths (different colored curves). The black dashed line is the theoretical impedance and the solid colored lines are obtained from the breath-by-breath ZVV analysis. Curves with different colors represent cycles corresponding to different ∆T_b_. The *R* and *E* irrespective of ∆T_b_ are plotted in the insets with and without pressure correction (*∆P* and *∆Ps*, respectively). Percent errors are shown with respect to theoretical steady-state values for *R* (**C**) and *E* (**D**) as a function of *F*_*R*_ with (*∆Ps*) and without (*∆P*) transient pressure adjustment.

The extent to which the transients affected ZVV is summarized in Fig. 4. There was a systematic error in *R* that increased with ∆T_b_, the difference in time periods in consecutive breaths, resulting in an overestimation at low *F*_*R*_ but an underestimation at high *F*_*R*_ (Fig. 4A). The error in *E* was much smaller (Fig. 4B). However, applying our correction for transient pressures (*∆P*_*s*_) drastically reduced the error in *R* (Fig. 4C) from 19% to −1.9% for an *F*_*R*_ close to 10 breath/min and from −42% to +1.6% for an *F*_*R*_ close to 20 breath/min. Nevertheless, *E* was properly estimated using both techniques with an error less than 2% (Fig. 4D). The proposed correction revealed the same findings and error reduction when the parameters of the model were varied (Supplementary Table S4). Finally, we also simulated the case when the algorithm missed the correct starting point of a breath randomly by up to 2 points (corresponding to a maximum absolute time difference of 0.04 seconds using the clinical trial sampling frequency of 50 Hz). The effects of this was negligible on the final results (see Supplementary Fig. S3 in Supplement D).

## Discussion

In this study, we introduced ZVV, an approach for continuously estimating a subject’s *R* and *E* at physiological breathing rates and tidal volumes during VV. We demonstrated ZVV’s feasibility in a clinical setting as a diagnostic potential in patients with mild ARDS, in an experimental setting using a mouse model of ARDS, and through computational modeling to validate the accuracy of the measured values. Our analysis demonstrated that ZVV can accurately and continuously detect changes in functional lung mechanics even when they are minor, without requiring any patient or ventilator manipulations. Furthermore, our preliminary results suggest that VV has the potential to not only serve as a diagnostic tool, but to provide benefits to patients via lung recruitment during ARDS.

The main concept behind ZVV is to utilize the breath-by-breath variations in tidal volume and respiratory rate provided by VV. It has been shown that a standard waveform does not have sufficient energy at frequencies higher than the breathing rate to allow estimating resistance and elastance and may include nonlinearities which can distort estimates at the higher harmonics^46^. Thus, in general, it may not be possible to get further frequency information from a single cycle of CV or even VV beyond the breathing rate. In other words, the technique itself can be applied to data collected during CV, but it will only provide resistance and elastance values at a single tidal volume and frequency (cycle’s breathing rate). In contrast, ZVV on data from VV produces a spectrum of values with specific physiological information that may provide more insight to the clinician.

ZVV’s primary limitation is that it should be applied to ventilation-driven breath cycles with zero intrinsic PEEP (iPEEP) unless the esophageal pressure and iPEEP are measured and accounted for^47–50^. Otherwise, the patient’s spontaneous breathing (or muscular activity) and iPEEP will affect the estimated parameters. Nevertheless, because there are so many breath during a longer term ventilation, VV still provides ample data to apply the technique without the extra complications of accurately accounting for these. Notice, for example, that patient 3 has almost half the number of breaths as patient 1 but still provides smooth curves and estimates. If the patient regularly attempts to breathe, then measurement of esophageal pressure may be required to separate ventilator-drive from active muscle contributions. Some ventilators can also measure iPEEP, which can also be included in the analysis. In our study, the ventilator was not designed to detect patient respiratory effort or iPEEP, and thus related errors may have been included in the resistance and elastance estimates. However, we assumed that these were the same across time and affected all measurements equally.

A further limitation is that ZVV requires ventilators delivering VV. Adding this feature is practical, requiring only software modifications, and encouraging since VV provides continuous recording of mechanical parameters which are useful for tracking changes in lung function during any intervention or drug testing. This will also allow further investigation into VV’s possible potential of being clinically therapeutic.

Another limitation is attributed to the fact that ZVV analyzes the full breath including passive expiration. To examine how this may affect the results, a time domain analysis was also applied (see Supplement C for details). The main results demonstrated similar trends without major differences between the time-domain and the ZVV-derived mechanical parameters (Supplementary Fig. S1).

For the patients, we do not have a gold standard such as the FOT to which ZVV can be compared. Nevertheless, the results in mice comparing ZVV and OVW together with the computational modeling imply that ZVV provides accurate assessments of respiratory system parameters.

Finally, there have been several developments in monitoring *E* continuously in the time domain^2,51^. While these approaches assume a model structure, which may incorrectly represent respiratory mechanics, ZVV, being a frequency domain-based approach, requires no predefined model. Furthermore, since the essence of Fourier analysis is to separate a signal’s inherent frequencies, ZVV can reduce the signal processing time because it might not require filtering of the waveforms as is often done in time domain. This may allow live tracking of *R* and *E* of patients in the future. Compared to other frequency domain techniques (i.e. FOT), ZVV estimates *R* and *E* under physiological conditions since it utilizes the ventilator’s breathing waveform.

### Human Clinical Trial

From Fig. 2 as well as Table 1, it is observed that the data obtained from ZVV are smooth having small error bars with sufficient quality to allow fitting of parametric models. This is because the binning process and the breath selection criteria partition and average the data over a relatively long time period. Thus, abnormal breaths which might have been missed by the algorithm or due to the patient’s own attempts to breathe have little effect on the final *R* and *E* spectra. For example, it is worth noting that patient 5’s *E* spectrum is least smooth and this may be attributed to patient-ventilator asynchrony.

The decrease in *R* and *E* from T1 to T2 in the patients implies that VV induced an improvement in lung mechanics. Indeed, some patients showed minimal yet statistically significant improvements. Surprisingly, *R* in all patients, and *E* in most patients, increased with *V*_*T*_ for both T1 and T2. While the increase in *R* can be attributed to nonlinear flow dynamics in the airways, the increase of *E* is less expected, yet it has been reported previously in patients^52^. This suggests that some recruited regions may have been over ventilated as higher tidal volumes reach the more nonlinear upper acinar pressure-volume curve. Regardless of the extent and cause of improvement, the ability to monitor the response to treatment or changes in ventilation settings provides a quantitative personalized approach to clinical ventilation. As Table 1 demonstrates, even a small improvement (or deterioration) in respiratory mechanics can be detected by ZVV. This has important implications for treating ARDS patients. Future studies could benefit from ZVV by tracking responses to clinical maneuvers in ARDS such as surfactant therapy, PEEP optimization, or prone positioning.

The decrease in *R* and *E* with time signifies improvement in patient condition. It is likely that VV itself induced this improvement for the following reasons. First, the study was administered when patients were in a stable condition and VV was always preceded by CV. Therefore, while statistically speaking improvement and deterioration would be equally likely, all 5 patients showed a decrease in both *R* and *E*. Second, previous studies provided evidence of improvement in lung mechanics, gas exchange, as well as reduction in ventilator-induced lung injury following VV compared to CV in both healthy and injured lungs of many animal models including pigs, mice, rats, and sheep^24–37^. Clinically, VV was also shown to improve gas exchange and respiratory mechanics^53^, improve patient-ventilator synchrony^54^, and reduce inflammatory response^55^. The present results support that VV could also improve lung physiology in human patients with mild ARDS. The most likely mechanism by which VV improves lung mechanics is recruitment^23^ and enhanced surfactant secretion^31,32^. To our knowledge, only one study applied VV in human subjects during abdominal surgery which did not result in any physiological improvement^56^; however, the subjects had normal lungs and the results are similar to our mouse data before lavage. Another possibility is that the ventilation time and/or settings were suboptimal for improvements to occur. Regardless, our findings provide preliminary results that encourage further investigation into the therapeutic effects of VV in human subjects. For future application of ZVV in human patients, VV’s utility could be extended from a potential treatment strategy into a powerful diagnostic tool as well. This is not the first study to propose VV as a diagnostic tool. Smith & Bates^57^, through the use of modeling, demonstrated that breath-to-breath variations inherent to VV are adequate for a numerical optimization algorithm that captures lung recruitment dynamics and derecruitment in healthy and injured mice. Their findings support VV’s ability to identify the extent of lung injury in ARDS. The real-time coupling of such models with ZVV could provide further insight into lung mechanics and optimized ventilation settings.

### Mouse experiments

Thammanomai *et al*.^33^ reported that *E* remained fairly constant during VV while it linearly increased with time during CV in mice subjected to acute lung injury *via* HCl instillation. Our ZVV findings are in agreement with that study. In particular, they showed that *E* remained fairly constant after ~15 minutes of VV. Supplementary Fig. S2 plots the ZVV time binned data before and after lavage. As in their experiments, our data also show that *E* reached a plateau only after lavage and specifically after about 5 minutes of ventilation in mice 1, 2 and 5. More importantly, there is a minor but statistically significant improvement from T1 to T2 after lavage (Supplementary Table S3). Unlike the patients, the therapeutic effects of VV are more apparent in mice since before lavage there was a slight increase in *E* attributed to progressive derecruitment, but following lavage there was a decrease indicating VV-induced recruitment. Similar to the patients, *R* mainly increased with increasing *V*_*T*_. However, opposite to the patient data, *E* decreased with increasing *V*_*T*_ suggesting in this case either alveolar recruitment or inverse tidal volume dependence^58,59^.

We also found differences between *R* and *E* from ZVV and OVW; however, only the former was significant. The OVW provides instantaneous results whereas the ZVV produces an average across many breaths. A more likely reason for the difference in *R* is due to how nonlinearities contribute to the FOT during OVW and ZVV. Higher frequency data in the OVW approach are estimated from the OVW’s small-amplitude components. In contrast, values from ZVV are obtained at much larger breathing amplitudes. This supports the idea that different techniques result in different assessments^60^ and therefore ZVV might be more precise since it assesses *R* and *E* at breathing frequencies.

### Computational Modeling

Calculating *Z* using Fourier analysis requires that the system be in a steady state which is not the case due to cycle-to-cycle variations leading to transients during VV. After accounting for the transients through a simple linear correction in pressure, the error was reduced by an order of magnitude. The correction is simple and can be carried out automatically. Furthermore, based on our modeling simulations (Fig. 4A), it can be seen that simply discarding those breaths from the calculations that have an *F*_*R*_ much larger or smaller compared to the previous cycle will significantly reduce the bias due to transients. However, for *E*, the difference was consistent and negligible even without correction (<2.5% in either case). Furthermore, *E* is more often used to guide mechanical ventilation since it reflects recruited alveolar units^2,12,13,61^. The frequency dependence of *E* in turn can reflect lung heterogeneity^62^ which might also provide useful information on how to set ventilation parameters. Hence, the frequency spectrum of *E* could be of significant relevance to clinicians, while the error in *R* may not be of concern in clinical applications.

## Conclusion

We have introduced ZVV, a technique to assess respiratory mechanics around the breathing frequencies in mechanically-ventilated subjects using Variable Ventilation. The ZVV approach is an accurate, experimentally and clinically-practical, and a personalized diagnostic tool during Variable Ventilation. This method also provided preliminary results that Variable Ventilation may improve lung mechanical conditions in patients with mild acute respiratory distress syndrome, and thus encourages further investigation.

## Supplementary information

Supplementary information.

## Data Availability

The datasets generated during and/or analyzed during the current study are available from the corresponding author on reasonable request.
