# Supplementary material for: Tracking respiratory mechanics around natural breathing rates via variable ventilation
Source: Sci Rep. 2020 Apr 21;10:6722. doi: 10.1038/s41598-020-63663-8 (PMC7174375; doi:10.1038/s41598-020-63663-8)
Supplement: Supplementary file 1 — Supplementary information. [file 41598_2020_63663_MOESM1_ESM.docx]

**Online Data Supplement**

**Tracking respiratory mechanics around natural breathing rates via variable ventilation**

Samer Bou Jawde^1^, PhD; Allan J. Walkey^2^, MD; Arnab Majumdar^1^, PhD; George T. O’Connor^2^, MD; Bradford J. Smith^3^, PhD; Jason H.T. Bates^4^, PhD; Kenneth R. Lutchen^1^, PhD; and Béla Suki^1^, PhD

Affiliation: ^1^Department of Biomedical Engineering, Boston University, Boston, MA; ^2^Department of Medicine, Pulmonary, Allergy, Sleep, & Critical Care Medicine, Boston University, Boston, MA; ^3^Department of Bioengineering, University of Colorado Denver | Anschutz Medical Campus, Aurora, CO; ^4^Pulmonary/Critical Care Division, University of Vermont, Burlington, VT

Supplement A: Forced oscillation technique

In 1956, DuBois et al.^1^ published the first respiratory impedance (*Z*) measurements using the forced oscillation technique (FOT) in human subjects. *Z*, defined as the ratio of airway opening pressure to flow in the frequency domain, was reported over a frequency range of 2 to 15 Hz. It became gradually evident that *Z* exhibits strong frequency dependence at low frequencies surrounding the spontaneous respiratory rate (*F_R_*: 0.1-0.3 Hz)^2–6^. For example, it is well accepted that *Z* calculated around spontaneous breathing rates is sensitive to alterations in lung properties including heterogeneity of disease which is relevant for clinical decision making^7,8^. Current FOT approaches, however, measure *Z* utilizing low amplitude pressure oscillations at frequencies far above *F_R_*, usually between 2 and 32 Hz^9,10^. Thus, these FOT measurements do not provide an assessment of lung function at *F_R_* potentially limiting insight into the physiological condition of the lung. Furthermore, traditional FOT measurements include several breathing cycles and are applied only periodically^10,11^. Moreover, in conscious patients, the FOT also requires the patient’s cooperation throughout the measurement. While there was some success in measuring low-frequency *Z*^12–14^, it has remained difficult to translate FOT into clinical use and had rarely been utilized in mechanically ventilated patients^12,13^. Our new approach, ZVV, obviates the need for specialized FOT equipment or ventilation methods that were developed in the past^2,10,13,15,16^ and unlike FOT provides physiologically relevant data by utilizing a relatively new approach to mechanical ventilation known as Variable Ventilation (VV)^17^. The essence of VV is that physiological values of tidal volume (*V_T_*) and *F_R_* vary on a breath-by-breath basis. Since each breath has a distinct amplitude and frequency, VV has the potential to explore lung function at multiple and physiologically relevant frequencies and amplitudes when combined with ZVV.

Since ZVV is measured utilizing the ventilator’s breathing waveform it estimates respiratory system impedance under more physiological conditions than the small-amplitude FOT. Furthermore, in contrast to the FOT and the optimal ventilator waveform (OVW, Supplement B) approaches, ZVV derives the frequency dependence of respiratory resistance (*R*) and elastance (*E*), calculated from the real and imaginary part of impedance respectively, from hundreds or thousands of breaths with varying *F_R_*. Additionally, since ZVV evaluates *Z* only at the fundamental frequencies of the ventilator waveform, *R* and *E* are minimally affected by nonlinearities that often hinder the FOT to produce smooth impedance spectra superimposed on breathing^15^. Finally, binning *R* and *E* according to *V_T_* also allows studying the effects of system nonlinearities at the physiological breathing rates.

A disadvantage of ZVV in comparison to FOT, is that each value of *R* and *E* is derived from a single cycle which means that in contrast to the FOT, frequency dependence of *R* and *E* cannot be obtained quickly. Furthermore, unlike the FOT in which the respiratory system is actively driven throughout the full cycle, ZVV analyzes the full breath including the passive expiration. However, the latter was shown to have no effect on the estimated *R* and *E* (see Supplement C).

Supplement B: Optimal Ventilation Waveform (OVW) analysis

The OVW^18^ is based on the forced oscillation technique (FOT, see Supplement A)^1^ and provides a composite forced oscillatory wave form at the airway opening with a peak-to-peak pressure amplitude that generates a volume signal matching the tidal volume delivered by the ventilator during mechanical ventilation. The frequencies present in the OVW waveform allow the computation of respiratory impedance (*Z*) as the complex ratio of the pressure-flow cross-power spectrum and the flow auto-power spectrum. The data obtained from OVW can then be used to fit the constant-phase model written as^7,19^:

$Z=R_{N}+I_{aw}j+\frac{G-jH}{\omega_{n}^{\alpha}}$ Eq. (B.1)

where *R_N_*, *I_aw_*, *G*, and *H* are the Newtonian resistance, airway inertance, tissue damping, and tissue elastance, respectively, $\alpha=\frac{2}{\pi}atan(\frac{H}{G})$ and $\omega$is the circular frequency (rad/s). Note that the circular frequency $\omega$ is normalized with $\omega_{0}=1$ radian so that $\omega_{n}=\frac{\omega}{\omega_{0}}$ is used in Eq. (B.1) in order to obtain meaningful units for *G* and *H*^20^. For the range of frequencies applied,$I_{aw}\approx0$. Thus, the equivalent 2-element resistance (*R*) and elastance (*E*) at the mean normalized frequency $\omega_{m}$ are written as:

$R=R_{N}+\frac{G}{\omega_{m}^{\alpha}}$ Eq. (B.2)

$E=H\omega_{m}^{1-\alpha}$ Eq. (B.3)

The constant-phase parameters were used to calculate the equivalent *R* and *E* values from Eqs. (B.2) and (B.3), respectively.

Supplement C: Time domain analysis

Motivation

During mechanical ventilation, the inspiratory cycle is driven by the ventilator, while expiration is a passive process. The ZVV approach, however, analyzes the full breath unlike the forced oscillation technique (FOT, Supplement A) in which the respiratory system is actively driven throughout the full cycle. In order to investigate the extent to which the respiratory resistance (*R*) and (*E*) values are influenced by passive expiration in ZVV, we also determined *R* and *E* in the time domain both from inspiration only and the full cycle by fitting the pressure-flow data using a 2-element *R*-*E* in-series model. This approach resulted in values for *R* and *E* similar to those obtained from ZVV and showed similar trends across time.

Methods

Using the breath-by-breath pressure (*P*), volume (*V*) and flow ($\dot{V}$) time data, *R*, *E*, and an offset pressure (*P_o_*) were estimated using linear regression applied to single compartment model of the following form:

$P\left( t \right)=R\dot{V}(t)+EV(t)+P_{o}$ Eq. (C.1)

To find *R* and *E* for the full breath, the entire period of each breath was used, whereas for inspiratory-only fitting, the inspiratory phase of the breath was used. The values of *R* and *E* were then binned as a function of time for both the human patients as well as the mice (Supplementary Figs. S1 & S2, respectively).


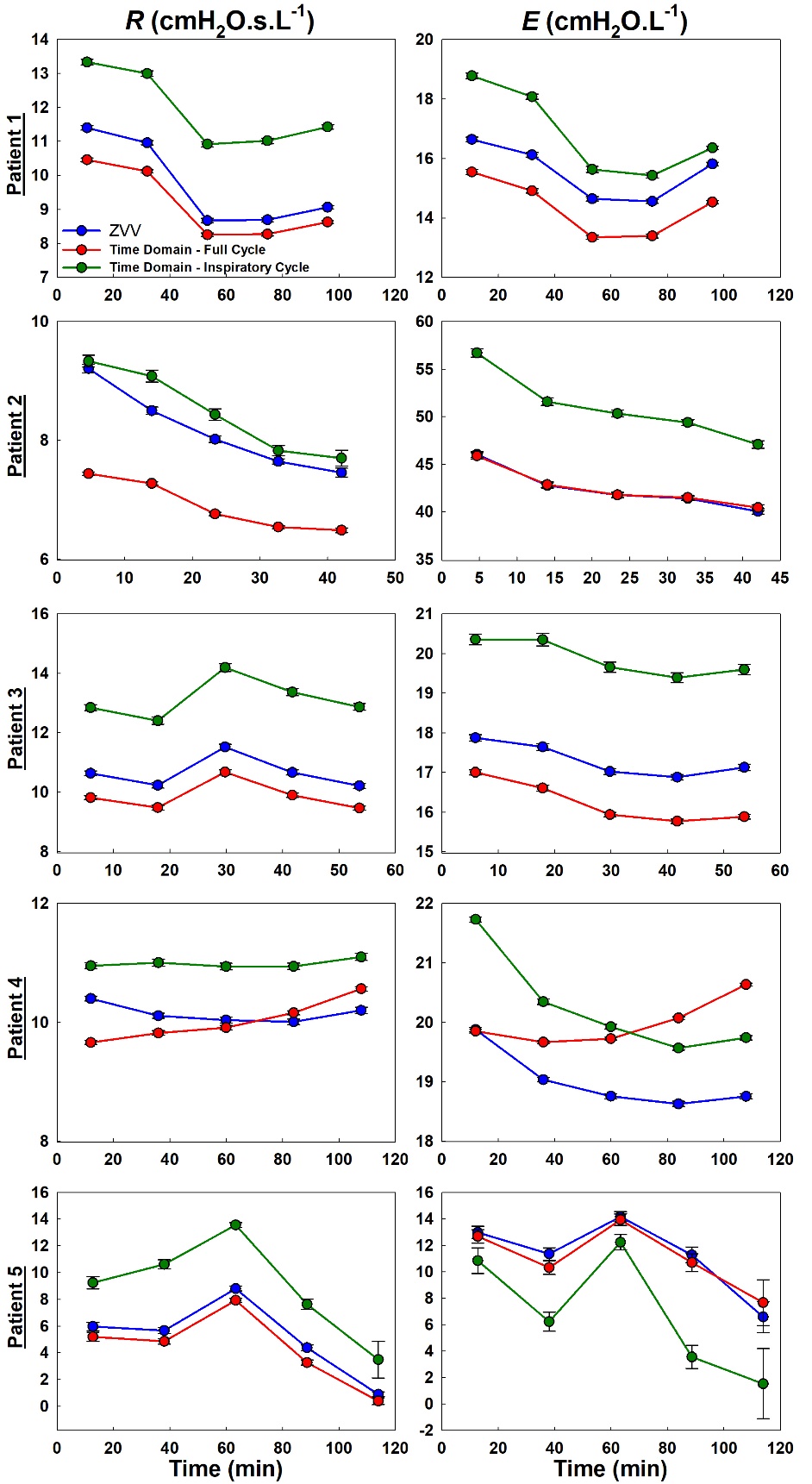


**Figure S1:** Time binned human data. Fitting full cycle or inspiratory cycle in time domain shows similar values and trends to ZVV.


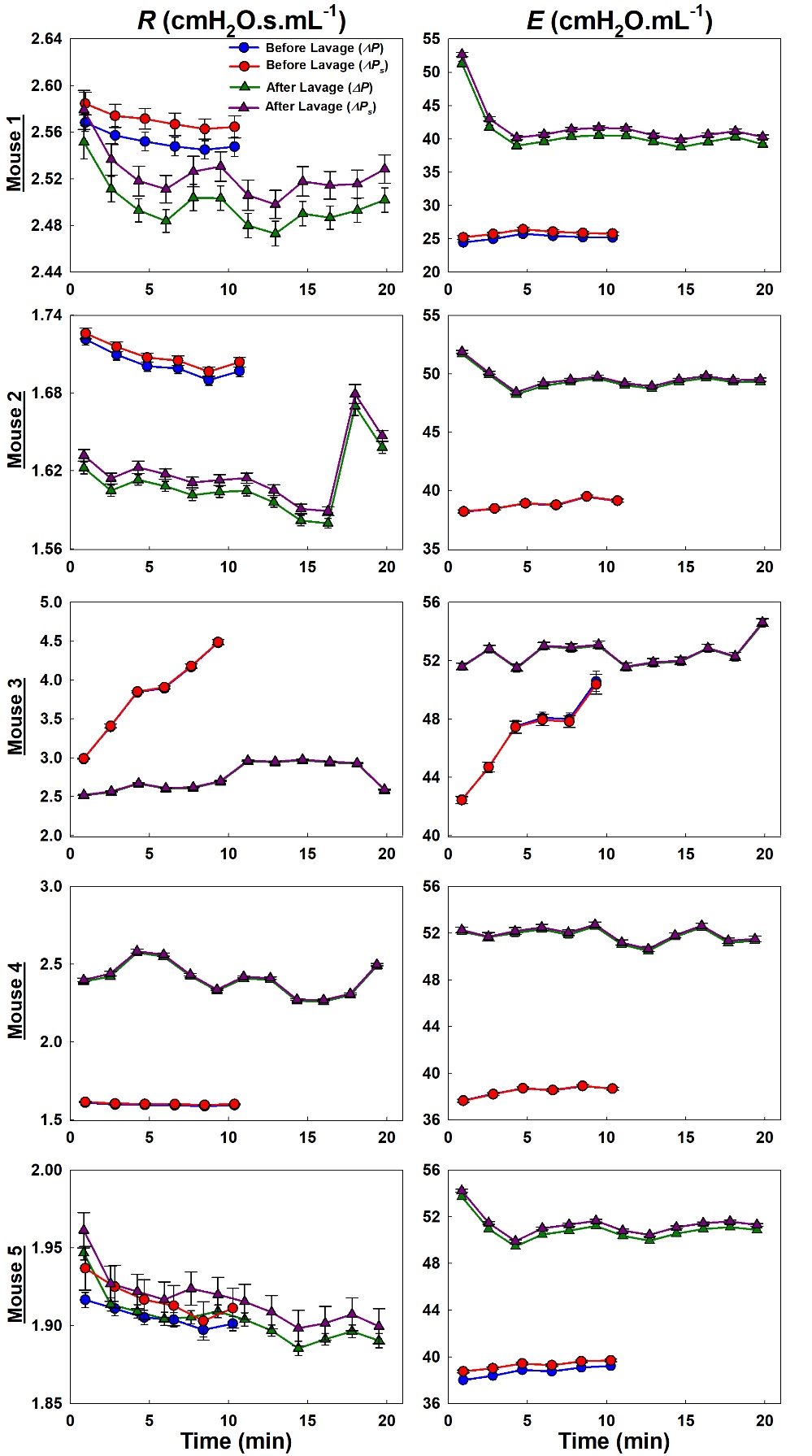


**Figure S2:** Time domain mouse data using ZVV with ∆P_s_ and ∆P. There is an underestimation of the values with ∆P but the difference is minor.

Results

In general, the inspiratory-only analysis provided higher values. However, the *R* and *E* values in time (inspiratory-only and full cycle) and frequency (ZVV) domain were generally close to each other and showed similar trends across time. It is important to note that the advantage of binning the data as a function of breathing frequency is that it provides frequency spectra of *R* and *E* which is not obtained when the data is binned in time. However, the ZVV technique can be used to bin data as a function of time as well when needed. Furthermore, the binning can be done as function of frequency (primary bin) and tidal volume (secondary bin) that can reveal the nonlinear properties of the respiratory system for a particular tidal volume at a specific frequency.

Supplement D: Computational study


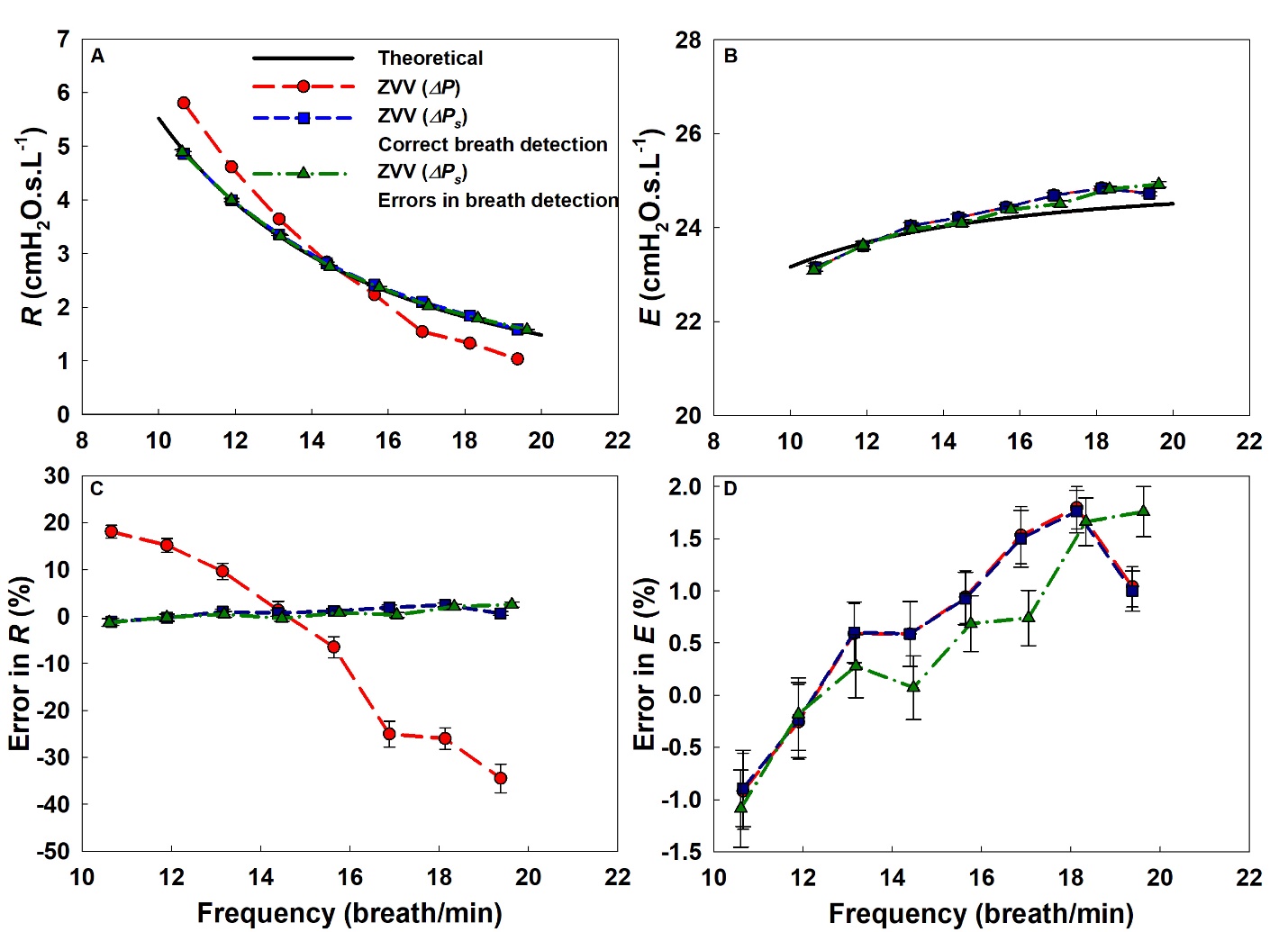


**Figure S3:** Effects of breath detection error on simulated R and E. The correct breath starting point was randomly shifted by any value from -2 to 2 (including zero corresponding to no error). ZVV was then applied to the unadjusted pressure (ΔP), adjusted pressure (ΔP_s_) with correct breath detection, and adjusted pressure with the shifted error for calculating A) R and B) E. C) and D) show the respective errors of R and E. Errors in detecting the beginning of the breath resulted in estimations very close to the correct breath detection.

References

1. DuBois, A. B., Brody, A. W., Lewis, D. H. & Burgess, B. F. Oscillation Mechanics of Lungs and Chest in Man. *J. Appl. Physiol.* **8**, 587–594 (1956).

2. Hantos, Z., Daróczy, B., Suki, B., Galgóczy, G. & Csendes, T. Forced oscillatory impedance of the respiratory system at low frequencies. *J. Appl. Physiol. Bethesda Md 1985* **60**, 123–132 (1986).

3. Fredberg, J. J. & Stamenovic, D. On the imperfect elasticity of lung tissue. *J. Appl. Physiol.* **67**, 2408–2419 (1989).

4. Fredberg, J. J., Keefe, D. H., Glass, G. M., Castile, R. G. & Frantz, I. D. Alveolar pressure nonhomogeneity during small-amplitude high-frequency oscillation. *J. Appl. Physiol.* **57**, 788–800 (1984).

5. Bates, J. H. *et al.* Interrupter resistance elucidated by alveolar pressure measurement in open-chest normal dogs. *J. Appl. Physiol.* **65**, 408–414 (1988).

6. Lutchen, K. R., Hantos, Z. & Jackson, A. C. Importance of low-frequency impedance data for reliably quantifying parallel inhomogeneities of respiratory mechanics. *IEEE Trans. Biomed. Eng.* **35**, 472–481 (1988).

7. Hantos, Z., Daroczy, B., Suki, B., Nagy, S. & Fredberg, J. J. Input impedance and peripheral inhomogeneity of dog lungs. *J. Appl. Physiol.* **72**, 168–178 (1992).

8. Rouby, J. J., Lu, Q. & Goldstein, I. Selecting the Right Level of Positive End-Expiratory Pressure in Patients with Acute Respiratory Distress Syndrome. *Am. J. Respir. Crit. Care Med.* **165**, 1182–1186 (2002).

9. LaPrad, A. S. & Lutchen, K. R. Respiratory impedance measurements for assessment of lung mechanics: focus on asthma. *Respir. Physiol. Neurobiol.* **163**, 64–73 (2008).

10. Oostveen, E. *et al.* The forced oscillation technique in clinical practice: methodology, recommendations and future developments. *Eur. Respir. J.* **22**, 1026–1041 (2003).

11. Kim, C. W., Kim, J. S., Park, J. W. & Hong, C.-S. Clinical Applications of Forced Oscillation Techniques (FOT) in Patients with Bronchial Asthma. *Korean J. Intern. Med.* **16**, 80–86 (2001).

12. Csorba, Z. *et al.* Capnographic Parameters in Ventilated Patients: Correspondence with Airway and Lung Tissue Mechanics. *Anesth. Analg.* **122**, 1412 (2016).

13. Navajas, D. & Farré, R. Forced oscillation assessment of respiratory mechanics in ventilated patients. *Crit. Care* **5**, 3 (2001).

14. Babik, B., Asztalos, T., Peták, F., Deák, Z. I. & Hantos, Z. Changes in Respiratory Mechanics During Cardiac Surgery. *Anesth. Analg.* **96**, 1280 (2003).

15. Suki, B. & Lutchen, K. R. Pseudorandom signals to estimate apparent transfer and coherence functions of nonlinear systems: applications to respiratory mechanics. *IEEE Trans. Biomed. Eng.* **39**, 1142–1151 (1992).

16. Daróczy, B. & Hantos, Z. Generation of optimum pseudorandom signals for respiratory impedance measurements. *Int. J. Biomed. Comput.* **25**, 21–31 (1990).

17. Lefevre, G. R., Kowalski, S. E., Girling, L. G., Thiessen, D. B. & Mutch, W. A. Improved arterial oxygenation after oleic acid lung injury in the pig using a computer-controlled mechanical ventilator. *Am. J. Respir. Crit. Care Med.* **154**, 1567–1572 (1996).

18. Lutchen, K. R., Yang, K., Kaczka, D. W. & Suki, B. Optimal ventilation waveforms for estimating low-frequency respiratory impedance. *J. Appl. Physiol. Bethesda Md 1985* **75**, 478–488 (1993).

19. Hantos, Z., Suki, B., Csendes, T. & Daroczy, B. Constant-phase modelling of pulmonary tissue impedance. *Bull Eur Physiopathol Respir* **23**, (1987).

20. Brewer, K. K. *et al.* Lung and alveolar wall elastic and hysteretic behavior in rats: effects of in vivo elastase treatment. *J. Appl. Physiol. Bethesda Md 1985* **95**, 1926–1936 (2003).

Tables

Supplementary Table S1: Patient biographic data.

| **Patient** | **Age** | **Height (cm)** | **Weight (kg)** | **Ideal Body Weight (kg)** | **BMI** | **Respiratory Failure etiology** | **APACHE II*** | **LIS**** |
| --- | --- | --- | --- | --- | --- | --- | --- | --- |
| 1 | 38 | 187 | 89 | 80 | 25.5 | pneumonia | 3 | 1 |
| 2 | 61 | 179 | 68.2 | 71 | 21.3 | pneumonia | 10 | 1 |
| 3 | 31 | 186 | 79.2 | 77.1 | 22.9 | nonpulmonary-sepsis | 29 | 2 |
| 4 | 63 | 170 | 119.9 | 66.1 | 41.5 | pneumonia | 22 | 2 |
| 5 | 41 | 160 | 61.5 | 56.9 | 24.0 | pneumonia | 20 | 1 |
| *APACHE II: Acute Physiology And Chronic Health Evaluation II ** LIS: Lung Injury Score | | | | | | | | |
|  |  |  |  |  |  |  |  |  |

Supplementary Table S2: Mice summary results.

| ***R* - Before Lavage** | | | | | | | |
| --- | --- | --- | --- | --- | --- | --- | --- |
|  |  | **T1** | | | **T2** | | |
| **Mouse** | **Time (min)** | **Breath Number** | **Mean** | **Standard Error** | **Breath Number** | **Mean** | **Standard Error** |
|  |  |  |  |  |  |  |  |
|  |  |  |  |  |  |  |  |
| **1** | 11.4 | 1220 | 2.56 | 0.005 | 1213 | 2.55 | 0.005 |
| **2** | 11.7 | 1245 | 1.71 | 0.002 | 1219 | 1.70 | 0.002 |
| **3** | 10.2 | 983 | 3.41 | 0.015 | 969 | 4.16 | 0.017 |
| **4** | 11.3 | 1211 | 1.60 | 0.002 | 1195 | 1.59 | 0.002 |
| **5** | 11.2 | 1189 | 1.91 | 0.003 | 1174 | 1.90 | 0.003 |
| ***R* - After Lavage** | | | | | | | |
|  |  | **T1** | | | **T2** | | |
| **Mouse** | **Time (min)** | **Breath Number** | **Mean** | **Standard Error** | **Breath Number** | **Mean** | **Standard Error** |
|  |  |  |  |  |  |  |  |
|  |  |  |  |  |  |  |  |
| **1** | 10.4 | 1097 | 2.52 | 0.007 | 1124 | 2.49 | 0.006 |
| **2** | 10.3 | 1083 | 1.61 | 0.003 | 1068 | 1.63 | 0.003 |
| **3** | 10.4 | 1094 | 2.57 | 0.004 | 881 | 2.84 | 0.007 |
| **4** | 10.1 | 1072 | 2.46 | 0.007 | 1055 | 2.34 | 0.005 |
| **5** | 10.2 | 1090 | 1.92 | 0.003 | 1062 | 1.89 | 0.002 |
| ***E* - Before Lavage** | | | | | | | |
|  |  | **T1** | | | **T2** | | |
| **Mouse** | **Time (min)** | **Breath Number** | **Mean** | **Standard Error** | **Breath Number** | **Mean** | **Standard Error** |
|  |  |  |  |  |  |  |  |
|  |  |  |  |  |  |  |  |
| **1** | 11.4 | 1220 | 25.05 | 0.162 | 1213 | 25.27 | 0.159 |
| **2** | 11.7 | 1245 | 38.50 | 0.084 | 1219 | 39.04 | 0.078 |
| **3** | 10.2 | 983 | 44.87 | 0.210 | 969 | 48.86 | 0.294 |
| **4** | 11.3 | 1211 | 38.19 | 0.083 | 1195 | 38.62 | 0.077 |
| **5** | 11.2 | 1189 | 38.37 | 0.072 | 1174 | 38.97 | 0.071 |
| ***E* - After Lavage** | | | | | | | |
|  |  | **T1** | | | **T2** | | |
| **Mouse** | **Time (min)** | **Breath Number** | **Mean** | **Standard Error** | **Breath Number** | **Mean** | **Standard Error** |
|  |  |  |  |  |  |  |  |
|  |  |  |  |  |  |  |  |
| **1** | 10.4 | 1097 | 43.85 | 0.245 | 1124 | 39.53 | 0.195 |
| **2** | 10.3 | 1083 | 49.98 | 0.100 | 1068 | 49.26 | 0.091 |
| **3** | 10.4 | 1094 | 51.94 | 0.142 | 881 | 51.56 | 0.150 |
| **4** | 10.1 | 1072 | 51.94 | 0.166 | 1055 | 51.56 | 0.131 |
| **5** | 10.2 | 1090 | 51.39 | 0.100 | 1062 | 50.87 | 0.080 |

Supplementary Table S3: Multiple comparison test results for *E* within T before and after lavage.

| **Mean *E* (cmH_2_O.mL^-1^)** | | | | | | | | |
| --- | --- | --- | --- | --- | --- | --- | --- | --- |
| **Mouse** | **Before lavage** | | | **After lavage** | | | **p value (T1 vs T2)** | |
|  | **T1** | **T2** | **(T2-T1)/T1** | **T1** | **T2** | **(T2-T1)/T1** | **Before lavage** | **After lavage** |
| **1** | 25.05 | 25.27 | 0.88% | 43.85 | 39.53 | -9.85% | 0.462 | **<10^-5^** |
| **2** | 38.50 | 39.04 | 1.40% | 49.98 | 49.26 | -1.45% | **<10^-5^** | **<10^-5^** |
| **3** | 44.87 | 48.86 | 8.89% | 51.94 | 51.56 | -0.72% | **<10^-5^** | 0.60 |
| **4** | 38.19 | 38.62 | 1.11% | 51.94 | 51.56 | -0.73% | **0.0168** | **0.0004** |
| **5** | 38.37 | 38.97 | 1.56% | 51.39 | 50.87 | -1.02% | **<10^-5^** | **<10^-5^** |

Supplementary Table S4: Simulation Results. The correction procedure was tested for 15 sets of simulations while varying different parameters. The error on *E* was consistently low before and after the correction. Errors without pressure correction were very high for *R*; however, the correction method consistently reduced errors as high as an order of magnitude, and the maximum error was only 3.4%.

|  | ***R*** | | ***E*** | |
| --- | --- | --- | --- | --- |
|  | **Maximum Error (%)** | | **Maximum Error (%)** | |
| **Parameter Change*** | ***∆P*** | ***∆P_s_*** | ***∆P*** | ***∆P_s_*** |
|  |  |  |  |  |
| **Baseline**** | -41.9 | -1.9 | 1.50 | -1.38 |
| **2Fs** | -44.4 | 2.1 | 1.67 | 1.40 |
| **Fs/2** | -35.2 | 1.7 | 2.12 | 2.03 |
| **2N_b_** | -37.5 | -2.1 | 1.62 | -1.38 |
| **N_b_/2** | -40.9 | -2.3 | 1.48 | -1.14 |
| ***F_R_* = 12.5-17.5** | -16.2 | 1.3 | 1.09 | 1.08 |
| ***F_R_* = 7.5-22.5** | -44.7 | 2.6 | 2.02 | 1.96 |
| **MV = 5** | -38.5 | -2.2 | 1.56 | -0.96 |
| **MV = 10** | -36.2 | -1.9 | 1.45 | -1.30 |
| **1.25*R_1_*** | -47.0 | 1.4 | 1.08 | 1.07 |
| **0.75*R_1_*** | -31.8 | 3.4 | 2.41 | 2.36 |
| **1.25*E_1_*** | -31.8 | 2.3 | 1.72 | 1.69 |
| **0.75*E_1_*** | -45.9 | 1.7 | 1.42 | 1.39 |
| **1.25*E_2_*** | -37.8 | 1.4 | 1.38 | 1.24 |
| **0.75*E_2_*** | -39.5 | 2.2 | 1.67 | 1.64 |
| *Compared to baseline  **Baseline: Fs = 50 Hz, N_b_ = 500 breath, *F_R_*=10-20 breath/min, MV = 7.5 L/min, *R_1_* = 60 cmH_2_O.s.L^-1^ , *E_1_* = 20 cmH_2_O.L^-1^, *E_2_* = 5 cmH_2_O.L^-1^ | | | | |
